# Machine learning analysis to automatically measure response time of pharyngeal swallowing reflex in videofluoroscopic swallowing study

**DOI:** 10.1038/s41598-020-71713-4

**Published:** 2020-09-07

**Authors:** Jong Taek Lee, Eunhee Park, Jong-Moon Hwang, Tae-Du Jung, Donghwi Park

**Affiliations:** 1 Artificial Intelligence Application Research Section, Electronics and Telecommunications Research Institute (ETRI), Daegu, Republic of Korea; 2 Department of Rehabilitation Medicine, School of Medicine, Kyungpook National University, 807 Hoguk-ro, Buk-gu, Daegu, 41404 Republic of Korea; 3 Department of Rehabilitation Medicine, Kyungpook National University Hospital, Daegu, Republic of Korea; 4 Department of Physical Medicine and Rehabilitation, Ulsan University Hospital, University of Ulsan College of Medicine, 877, Bangeojinsunhwando-ro, Dong-gu, Ulsan, 44033 Republic of Korea

**Keywords:** Motility disorders, Dysphagia

## Abstract

To evaluate clinical features and determine rehabilitation strategies of dysphagia, it is crucial to measure the exact response time of the pharyngeal swallowing reflex in a videofluoroscopic swallowing study (VFSS). However, measuring the response time of the pharyngeal swallowing reflex is labor-intensive and particularly for inexperienced clinicians, it can be difficult to measure the brief instance of the pharyngeal swallowing reflex by VFSS. To accurately measure the response time of the swallowing reflex, we present a novel framework, able to detect quick events. In this study, we evaluated the usefulness of machine learning analysis of a VFSS video for automatic measurement of the response time of a swallowing reflex in a pharyngeal phase. In total, 207 pharyngeal swallowing event clips, extracted from raw VFSS videos, were annotated at the starting point and end point of the pharyngeal swallowing reflex by expert clinicians as ground-truth. To evaluate the performance and generalization ability of our model, fivefold cross-validation was performed. The average success rates of detection of the class “during the swallowing reflex” for the training and validation datasets were 98.2% and 97.5%, respectively. The average difference between the predicted detection and the ground-truth at the starting point and end point of the swallowing reflex was 0.210 and 0.056 s, respectively. Therefore, the response times during pharyngeal swallowing reflex are automatically detected by our novel framework. This framework can be a clinically useful tool for estimating the absence or delayed response time of the swallowing reflex in patients with dysphagia and improving poor inter-rater reliability of evaluation of response time of pharyngeal swallowing reflex between expert and unskilled clinicians.

## Introduction

**Figure 1:**
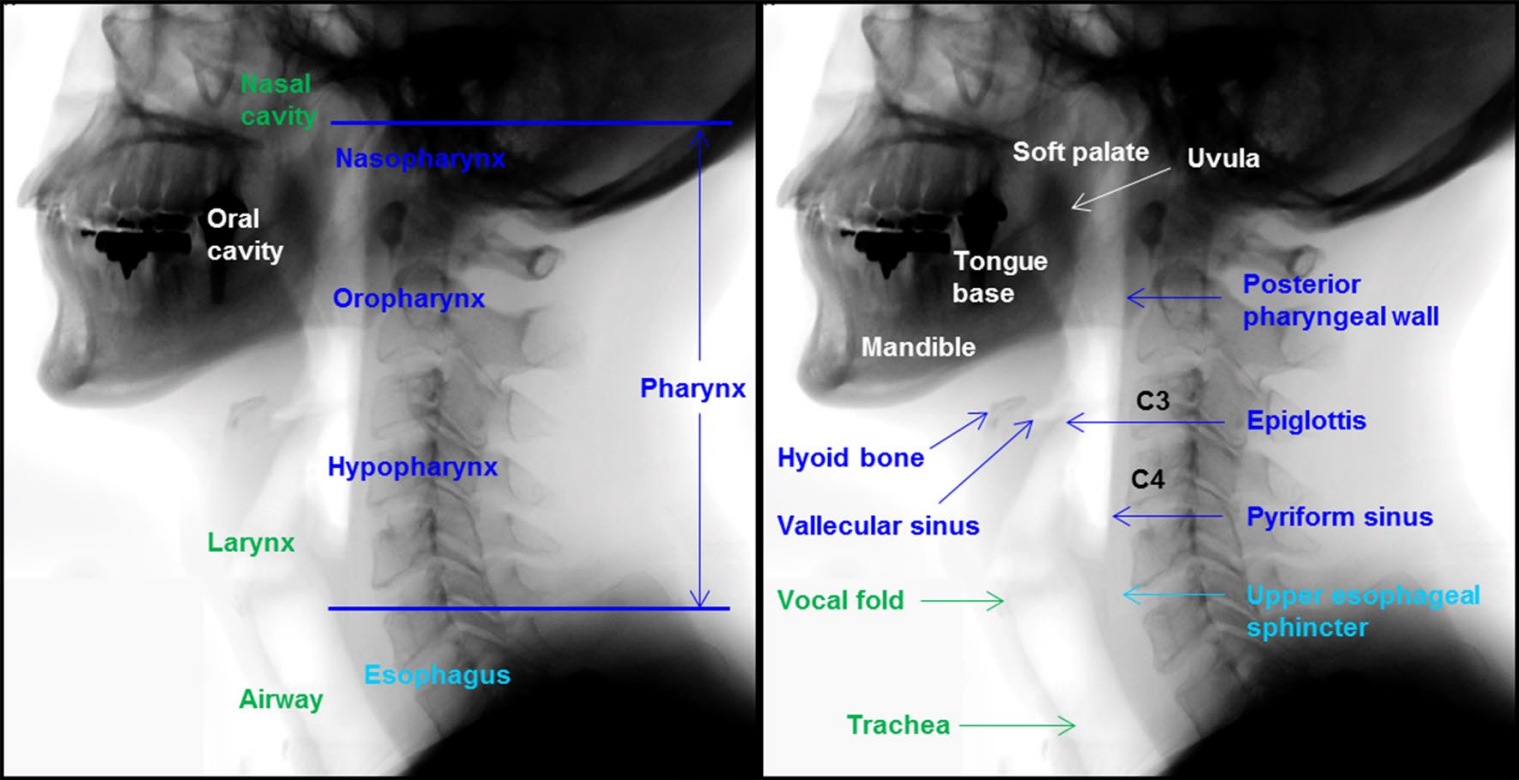
The anatomy associated with the swallowing process. Blue color indicates anatomical structures of pharynx related to swallowing, and green color indicates anatomical structures related to airways. White color indicates anatomical structures of oral cavity related to swallowing, and light blue color indicates anatomical structures of esophagus related to swallowing.

The process of swallowing includes the coordinated contraction and inhibition of the muscles of the tongue, pharynx, and esophagus by the central nervous system from the brain cortex to the brainstem^1^. The swallowing process is divided into three phases: oral, pharyngeal, and esophageal. The pharyngeal phase of swallowing is initiated when a food bolus (bolus) is moved from the oral cavity to the pharyngeal cavity^2^, and is initiated by a pharyngeal swallowing reflex, which is elicited unconsciously by stimulating receptive regions of the oropharynx such as soft palate and uvula (Fig. 1)^3^. The pharyngeal swallowing reflex is modulated by an input from the cerebral cortex and respiratory center and mediated in a reticular formation located in the brainstem^4^. This reflexive event requires coordination of the oropharynx and occurs to protect the airway during the swallowing process^3^. When food is passed from the oral cavity to the pharynx during swallowing, a pharyngeal swallowing reflex occurs, which causes the elevation of the larynx, preventing the aspiration of the food into the trachea.

In a clinical setting, absent or delayed response of the pharyngeal swallowing reflex is a critical sign and can cause aspiration of bolus before the beginning of the pharyngeal phase of the swallowing process, in patients with cerebrovascular and neurodegenerative diseases^5–7^. About 75% of stroke patients who presented with aspiration during swallowing showed delayed time of the pharyngeal swallowing reflex^5^. In addition, an initially absent or delayed swallowing reflex is an independent prognostic predictor of aspiration pneumonia in 6 months after a stroke^6^. Triggering pharyngeal swallowing reflex is a crucial phenomenon to protect the airway and prevent aspiration during swallowing^8^. In healthy people, the pharyngeal swallowing reflex occurs in less than 0.5 s^9^. Owing to the pharyngeal swallowing reflex being rapid, manual measurements of the reflexive time requires extensive clinical experience. A study demonstrated the poor inter-rater reliability of the measured time of pharyngeal swallowing reflex between an inexperienced and expert clinician^10^. Thus, it could be useful, especially for unskilled clinicians, to provide reliable response time estimation for pharyngeal swallowing reflex from raw VFSS video.

To precisely diagnose and quantitatively analyze clinical dysphagia, the videofluoroscopic swallowing study (VFSS) is currently considered the gold standard method^11^. Clinicians repeatedly examine quantitative and spatiotemporal parameters in a VFSS recorded video based on a frame-by-frame analysis^12^. Several software tools provide semi-automatic measurements of various parameters in a VFSS video^13,14^. However, to use these tools, clinicians have to mark the region of interest (ROI) in each frame. Meanwhile, the manual tracking of anatomical structures and movements in each frame is costly in terms of time and clinical expertise. With the recent advances in research on machine learning in the medical field, several methods of machine learning-based VFSS analysis have been reported. Using the single shot multi-box detector, one of state-of-the-art deep learning methods for object detection, Zhang et al.^15^ developed a tracking system for the detection of the hyoid bone. However, it is challenging to analyze motion or action in the VFSS videos using this method, because this method focuses on detection of the spatial region of a single image rather than on the analysis of a sequence of images from a video. To overcome this limitation, previous studies used the integrated 3-dimensional (3D) convolutional network^16,17^, a state-of-the-art video analysis method, for detection of the pharyngeal phase in a VFSS video without manual spatial annotations^16,17^. The detection of the pharyngeal phase is useful for shortening the VFSS examination time by the clinician by removal of unrelated frames. However, manual analysis of the pharyngeal phase requires determination of the status of the patients. Therefore, this study proposes a novel framework to automatically measure the response time of the pharyngeal swallowing reflex, one of the most critical indicators that can be directly used for deciding patient treatment.

While most of the state-of-the-art 3D convolutional networks including C3D^18^ and I3D^19^ take a video clip recording of at least 16 frames as input, the average frame length of the pharyngeal swallowing reflex is less than the frame length of a 3D convolutional network’s input clips. Owing to the short response time of the swallowing reflex that makes it difficult to train a 3D convolutional network, a novel data augmentation and training method is proposed.

The contribution of this study to clinical settings is threefold. First, our framework provides reliable response time estimation for pharyngeal swallowing reflex from raw VFSS video. Second, it can be helpful for all clinicians in determining normal, delayed, or absent swallowing reflex from a VFSS video. Third, it can provide clinical information for clinicians to decide rehabilitation strategies, such as the thermal-tactile stimulation that may lead to a more rapid triggering of pharyngeal swallow, for dysphagic patients with absent or delayed swallowing reflexes.

## Materials and methods

### Data collection

The VFSS data was collected from 27 participants who reported subjective swallowing difficulties and visited the outpatient clinic of the Department of Rehabilitation Medicine at Kyungpook National University Chilgok Hospital from March to May 2017. The participants were between 22 and 84 years old (mean age 64.9 ± 15.7 years) and included 21 males and 6 females. A subset of participants were healthy, aged over 65 years (N = 3, 11.1%); and the remaining participants were diagnosed with a central nervous system disease (N = 16, 59.2%), or a neuromuscular disease (N = 8, 29.6%). All experimental protocols of this study were approved by the Institutional Review Board at the Kyungpook National University Chilgok Hospital (No. KNUCH 2018-05-006). All methods were carried out in accordance with relevant guidelines and regulations. Informed consent was obtained from all subjects or, if subjects are under 18, from a parent and/or legal guardian.

**Figure 2:**
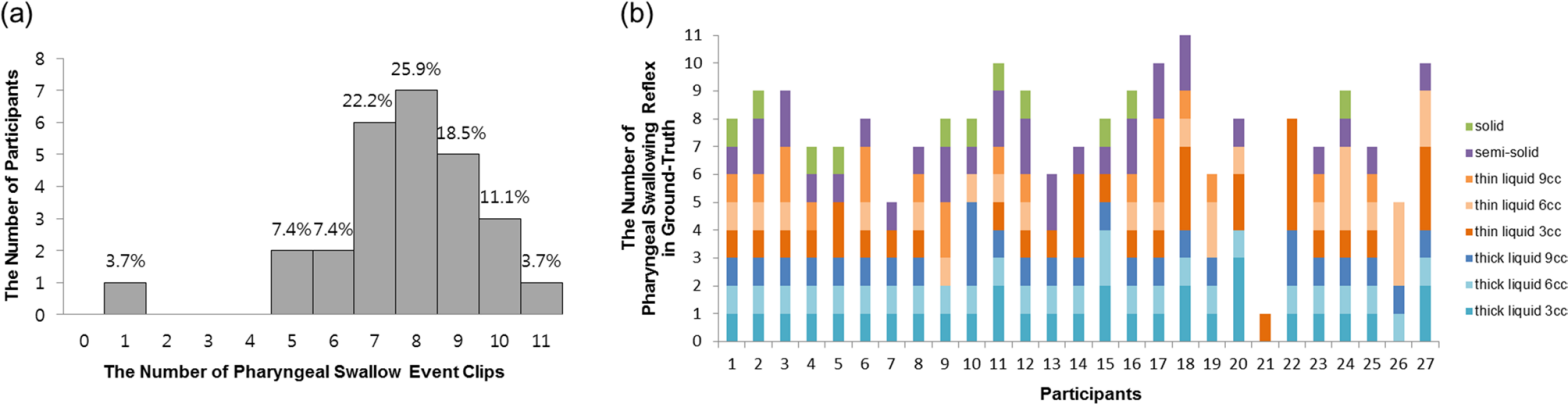
The distribution of pharyngeal swallowing events in a videofluoroscopic swallowing study. (**a**) The distribution of the number of participants according to the number of pharyngeal swallowing event clips, and (**b**) the distribution of the number of pharyngeal swallowing reflexes in 207 ground-truth datasets according to each participant.

Clinicians performed the VFSS procedure according to the standard manual guidelines^2^. During the VFSS procedure, the participant was seated upright in front of a fluoroscope, which was set to 30 frames per second. Each participant swallowed 8 substances mixed with diluted radiopaque barium (35% w/v): 3, 6, and 9 mL of curd-type yogurt (thick liquid); 3, 6, and 9 mL of water (thin liquid); semi-blended rice (semi-solid); and steamed rice (solid). The lateral view of the head and neck areas in the VFSS image were recorded by a camcorder (HDR-CX405X, SONY, Japan). From the 27 participants, 7 participants completed 8 pharyngeal swallowing events ingesting 8 substances during the VFSS. Nine participants completed more than 8 pharyngeal swallowing events owing to multiple swallows during engulfing of one substance. Eleven participants completed less than 8 pharyngeal swallowing events, as they did not completely swallow all substances owing to severe aspiration during the VFSS procedures (Fig. 2).

**Figure 3:**
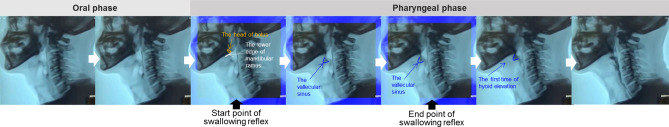
An example of ground-truth. The blue boundary boxes represent the ground-truth of the pharyngeal swallowing reflex. The starting point of the swallowing reflex is defined as the first video frame of the head of bolus reaching the lower edge of the mandibular ramus. The end point of the swallowing reflex is defined as the last video frame in which the head of the bolus reaches the vallecular sinus until the first time of hyoid bone elevation is triggered by pharyngeal swallowing.

Video clips of pharyngeal swallowing events of each substance were obtained at 15 frames per second (FPS). The 207 video segments of pharyngeal swallowing events were extracted from the raw recorded videos of the VFSSs using the 3D convolutional network-based automatic detection of the pharyngeal phase which was reported in our previous study^17^. Two expert physiatrists, who had analyzed the biomechanical parameters of the VFSS, independently evaluated and labeled the starting point and end point of the pharyngeal swallowing reflex in the video as the ground-truth. The starting point of a pharyngeal swallowing reflex is defined as the first video frame in which the head of the bolus reaches the lower edge of the mandibular ramus. The end point of the swallowing reflex is defined as the last video frame in which the head of the bolus reaches the vallecular sinus, until the first time of hyoid bone elevation is triggered by a pharyngeal swallow (Fig. 3)^2^. In case of a disagreement between the two experts, a consensus was reached through discussion. Consequently, we gathered 207 ground-truth data samples labeled with the response time of the pharyngeal swallowing reflex as shown in Fig. 3.

### Network architecture

**Figure 4:**
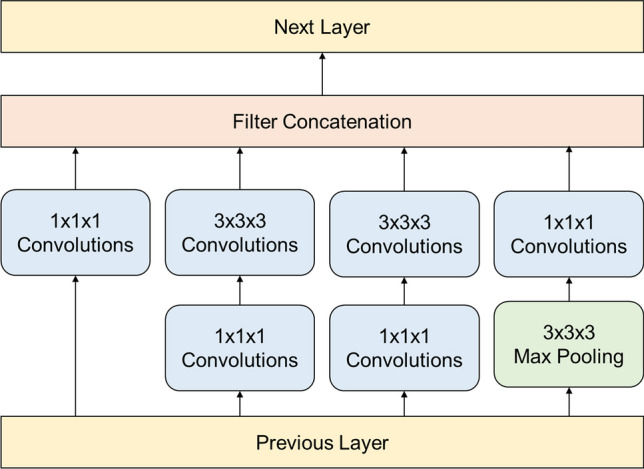
Inception module used in the Inception-V1 architecture (3D convolutional network) to reduce computational cost and avoid overfitting.

For our model, we considered the Inflated 3D Convolutional Network (I3D)^19^ as our front-end network architecture, because the I3D is one of the most successful action recognition methods in large-scale action recognition benchmarks^20^. Furthermore, it has been proven very effective in VFSS analysis, such as in pharyngeal phase detection^17^. While Lee et al*.*^17^ proposed a new network architecture design to improve the detection rate, the Inception-V1 achieved higher accuracy if its pre-trained weights were provided. In this study, we used a pre-trained Inception-V1 architecture as a base network to accelerate the training process. The Inception-V1 architecture contains four max-pooling layers, one average pooling layer, two convolutional layers, and nine inception modules. The inception module, which was designed to improve computational expense and overfitting, is a concatenation of four 3D convolutions with two different sizes of filters (Fig. 4). The I3D network of the Inception-V1 we used, was pre-trained by the Kinetics human video dataset^21^, which contains 400 human action classes and at least 400 video samples for each class. The time duration of input video for the pre-trained network was 2.56 s, which is similar to the video time duration of pharyngeal phase videos but much longer than videos of swallowing reflex events.

### Data augmentation for short interval event detection

The duration of the pharyngeal swallowing reflex in ground-truth, which varies from 0.067 to 0.867 s with a mean of 0.292 s, is much shorter than the interval of the entire pharyngeal phase. Hence, there would not be a sufficient number of frames containing the swallowing reflex to directly train the 3D convolutional network, which requires a large number of frames in each video to integrate temporal features from a longer temporal receptive field. For example, C3D^18^ and I3D^19^ are two of the most popular 3D convolutional networks, and they take a video clip of length 16 frames and 64 frames, respectively. Simply reducing the number of input frames can cause classification accuracy degradation. Zhang et al.^22^ showed that gesture classification accuracy decreased from 0.864 to 0.817 as the number of input frames decreased from 16 to 8.

**Figure 5:**
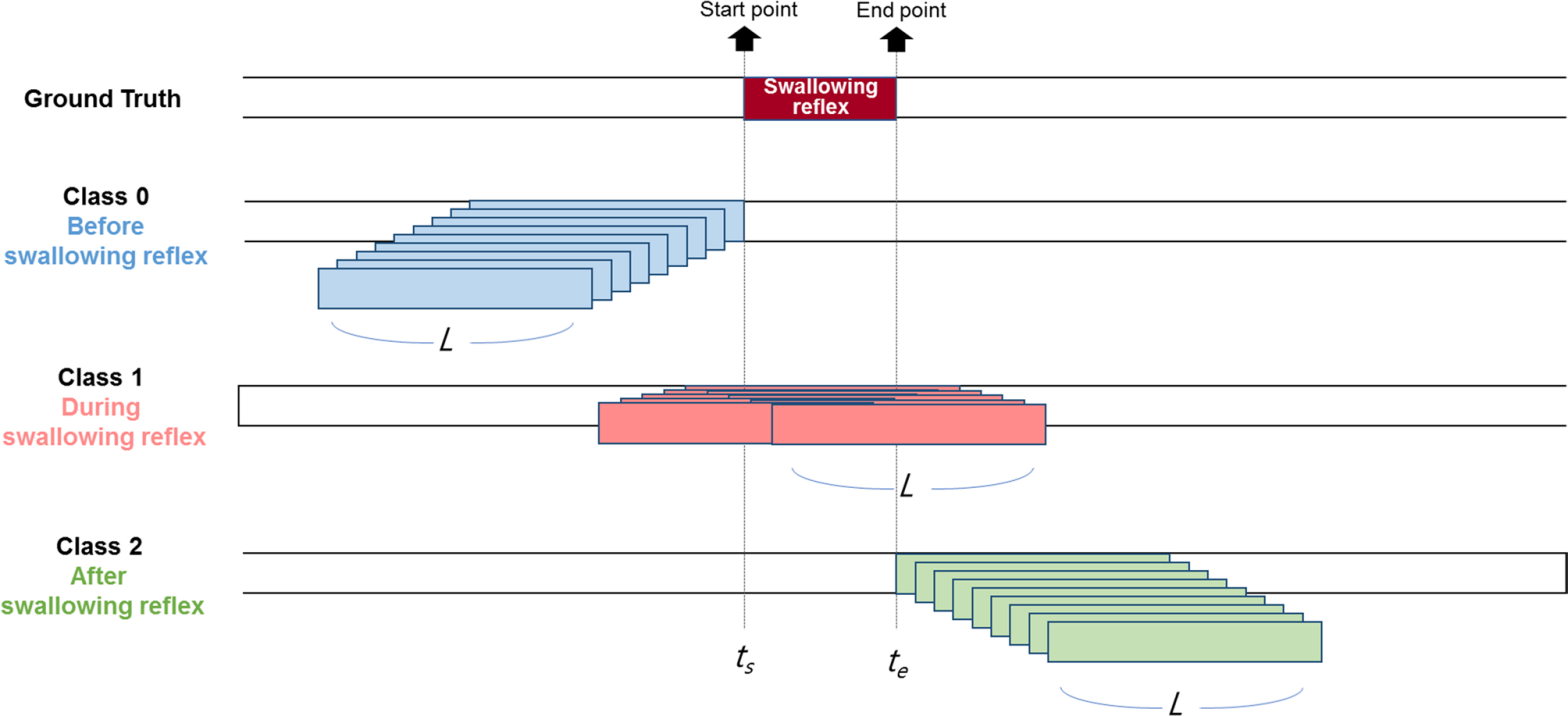
Generation of the three classes and classification of short interval events for training. Class 0, 1, and 2 represent before, during, and after swallowing reflex, respectively.

Therefore, instead of classifying whether a very short video clip is of the swallowing reflex, three classes related to the swallowing reflex were defined: classes 0, 1, and 2 indicate *before, during, and after the swallowing reflex*, respectively. As shown in Fig. 5, the total number of frames of a video clip for all classes is constant, *L*, and the beginning and ending frame index of the swallow reflex are defined as *t*_*s*_ and *t*_*e*_, respectively. For collecting training data of *before swallowing reflex* (Class 0), the interval [*t*_*s*_—*L*, *t*_*s*_] is determined, ending right before the beginning of the swallowing reflex. Then, 9 intervals of [*t*_*s*_—*L—i*, *t*_*s*_—*i*] are collected for *i* from 0 to 8. For collecting training data of *after swallowing reflex* (Class 2), the interval, [*t*_*e*_, *t*_*e*_ + *L*] is determined, starting immediately after the ending of the swallowing reflex. Subsequently, 9 intervals of [*t*_*e*_ + *i*, *t*_*e*_ + *L* + *i*] are collected for *i* from 0 to 8. For collecting *during swallowing reflex* (Class 1), the interval whose center is closest to the center of the swallowing reflex was first determined. Then, 9 intervals that were closest to the interval first found were collected, such as [(*t*_*s*_ + *t*_*e*_—*L*) / 2 + *i*, (*t*_*s*_ + *t*_*e*_ + *L*) / 2 + *i*] for *i* from -4 to 4. Therefore, we collected 27 samples (9 for each class) for training of the 3D convolutional network from a single annotated video clip of the swallowing reflex.

### Training and testing

The fivefold cross-validation was performed to demonstrate the generalization ability of our model. While leave-one-out cross-validation is approximately unbiased and takes almost an entire dataset as a training set, it is computationally expensive, as it requires fitting the model for each sample of the dataset^23^. In our study, the average time for training of the I3D model with our data is about 10 h on a machine equipped with a single Titan X graphic processing unit. Thus, it is time consuming to apply leave-one-out cross-validation. About 80% of the entire labeled swallowing reflex videos were used as the training set, and the rest were used as the testing set. For the testing dataset to consist of patients not included in the training dataset, we chose 5 to 6 patients out of the 27 participants and separated all of their swallowing reflex videos from the testing data. Consequently, 5 groups of testing data were generated, and the number of swallowing reflex events of the testing data varied from 40 to 42. The remaining videos of patients’ swallowing reflex were used as training data. After data augmentation, the number of samples for training the I3D model was about 4,500. The input video data contained 20 frames (*L* = 20) with 15 FPS, and each frame was resized to 224 × 224 pixels. Only an RGB stream was used as input stream because the performance gain from using both RGB and optical flow streams is small, and optical flow calculation is computationally expensive.

### Evaluation metrics

To evaluate the performance of detecting the response time of a pharyngeal swallowing reflex, the detection F-1 score and time error of the starting point and end point in the swallowing reflex were measured. The intersection over union (IOU) has been widely used as an evaluation metric for various tasks of detection. For detection of a pharyngeal swallowing reflex in pharyngeal phase clips, the IOU can be defined as the ratio of the frame length of intersection out of the frame length of union between predicted and ground truth time-predicates of the swallowing reflex^24^. A predicted detection is considered a True-Positive (TP) only if there is a ground-truth detection satisfying the condition that the IOU of the predicted detection and the ground-truth detection is larger than a threshold. A false-positive (FP) is a predicted detection if there is no ground-truth detection with an IOU larger than threshold. A false-negative (FN) is a true detection that was not predicted. Precision is the proportion of TPs out of all predicted detections (TP + FP). Recall is the proportion of TPs out of all ground-truth detections (TP + FN). The F-1 score is the harmonic mean of the precision and recall. Finally, the detection time error is calculated from the difference of the frame indexes of the starting point and end point of the swallowing reflex in between the TP and the ground-truth^24^.

## Results

### Reliability of measuring the time of pharyngeal swallowing reflex

To determine the inter-rater and intra-rater reliability, a VFSS video of 10 patients with dysphagia was used, and intra-class correlation coefficients (ICC) with corresponding 95% confidence interval (CI) were calculated. To evaluate intra-rater reliability, one examiner re-analyzed the measurement of the time of the swallowing reflex. Therefore, the time of the pharyngeal swallowing reflex in VFSS video was analyzed twice at different time points by an examiner blinded to clinical information. We achieved intra-rater reliability of ICC = 0.982 (CI: 0.972–0.989). In addition, to evaluate the inter-rater reliability, two examiners, blinded to clinical information and the results of the measurement by the other examiner, analyzed the measurement of the time of the pharyngeal swallowing reflex at different time points. We achieved inter-rater reliability of ICC = 0.968 (CI: 0.939–0.983).

### The results of machine learning process

**Figure 6:**
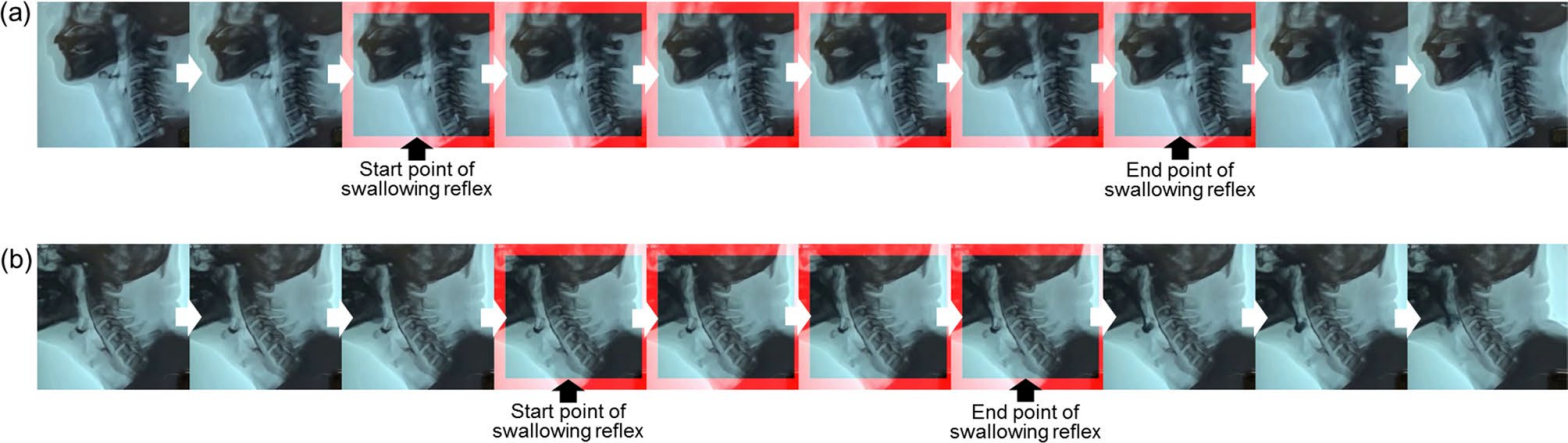
Two cases of true-positive predictive detection of swallowing reflex in patients with (**a**) motor neuron disease, and (**b**) Parkinson disease. The red boundary boxes represent true-positive predicted detection of swallowing reflex.

**Figure 7:**
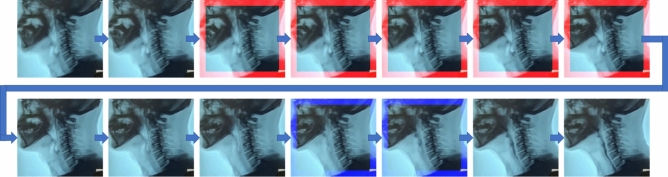
A case of missed detection. The red and blue boundary boxes represent predicted detections and ground-truth, respectively.

For the training set, the overall mean and standard deviation of the success rate of detection of *during pharyngeal swallowing reflex* (Class 1) of the 5 groups for cross-validation were 98.2% and 0.3%, respectively, and the average IOU was 0.537 (± 0.224). For unseen testing swallowing events, the overall mean and standard deviation of the success rate of detecting Class 1 in the 5 groups were 97.5% and 0.2%, respectively, and the average IOU was 0.573 (± 0.228). There were two cases of TP predicted detection in the response time of a swallowing reflex using this system, as shown in Fig. 6. A case of missed detection is shown in Fig. 7.

**Figure 8:**
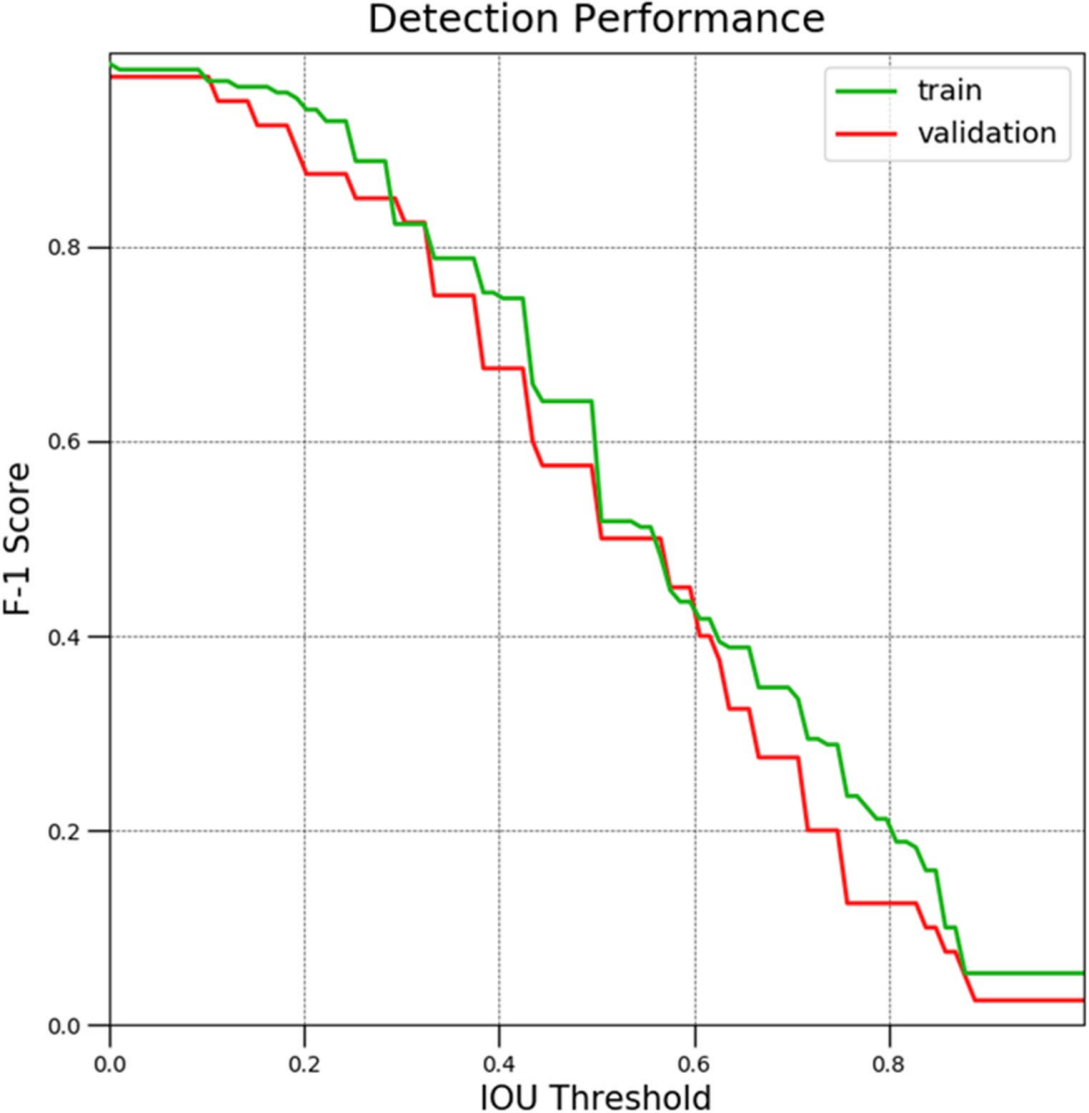
Free response operating characteristic curve of detection F-1 score and intersection over union (IOU) threshold.

Figure 8 shows the free response operating characteristic (FROC) curve of the detection F-1 score of Class 1 from group 1 (from the 5 groups) according to the IOU threshold in training and validation sets. With the IOU threshold at 0.2, the detection F-1 scores were 94.7% and 87.5% in the training and validation sets, respectively. With the IOU threshold at 0.4, the detection F-1 scores were 74.7% and 67.5% in the training and validation sets, respectively.

In the training set, the average difference between the TP and the ground-truth detection at the starting point and end point of the swallowing reflex was 0.149 and 0.081 s, respectively. In the validation set, the average difference between the TP and the ground-truth detection at the starting point and end point of the swallowing reflex was 0.210 and 0.056 s, respectively, as shown in Table 1.

**Table 1:** The performance of detecting response time of pharyngeal swallowing reflex.

	Training set	Validation set
Miss rate (%)	1.76	2.50
IOU (%)	53.7 ± 22.4	57.3 ± 22.8
**Time error (s)**		
Starting point of swallowing reflex	0.149 ± 0.113	0.210 ± 0.185
End point of swallowing reflex	0.081 ± 0.080	0.056 ± 0.060

Each cell represents mean ± standard deviation.

*IOU* intersection over union.

## Discussion

In this study, we proposed a novel framework of automatically detecting the response time for the pharyngeal swallowing reflex from VFSS video. The average success rates of detection of the class *during the swallowing reflex* for training and validation datasets were 98.2% and 97.5%, respectively. Kim et al.^10^ reported that, in a clinical setting, inter-rater reliability of the response time of the pharyngeal swallowing reflex between an inexperienced resident and expert physiatrist was low (ICC = 0.300; CI 0.250–0.351)^10^. However, our novel framework provided accurate and reliable results of time measurement of the swallowing reflex when compared with ground-truth labeled by two experts. Therefore, our framework can be used to provide considerable clinical information about dysphagic patients. Moreover, it may be utilized to measure various other spatiotemporal parameters in a VFSS, such as the laryngeal elevation and oral/pharyngeal transit time, even though they represent short time intervals in video clips.

Furthermore, our method demonstrated clinically meaningful results in measuring the response time of pharyngeal swallowing reflexes. The difference between the predicted response time of the swallowing reflex and the ground-truth was approximately 1–2.5 frames (0.067–0.167 s). A study reported that, in the VFSS, the normal value of the response time of the swallowing reflex was 0.21 ± 0.26 s in healthy young subjects and 0.53 ± 0.64 s in elderly subjects (≥ 65 year old)^25^. Our differences between predicted detection and ground-truth were within the standard deviation of swallowing reflex time in healthy subjects. Therefore, our framework is useful for diagnosing absent or delayed pharyngeal swallowing reflex in dysphagic patients. Moreover, it can be useful for clinicians deciding rehabilitation strategies that may trigger pharyngeal swallowing reflex, such as thermal-tactile stimulation.

To the best of our knowledge, there have been no previous studies on the use of machine learning analysis to measure the response time of the pharyngeal swallowing reflex despite its importance. As mentioned above, the detection of abnormality of pharyngeal swallowing reflex through measurement of swallowing reflection time is crucial for diagnosing dysphagia and evaluating its cause. However, there is considerable difficulty in measuring the response time of swallowing reflex by unskilled clinicians^10^. Therefore, measuring the pharyngeal swallowing reflex time using machine learning analysis can be helpful in identifying the cause of the swallowing disorder. Moreover, despite the advantage of the VFSS in being able to objectively observe the entire process of the swallowing process, the interpretation of the VFSS is complex and requires consideration of many other factors. To date, most VFSS analysis software programs have focused on tracking anatomical structures such as hyoid bones. However, in clinical settings, the interpretation of a VFSS is judged by considering not only hyoid bone motion but also other parameters such as the pharyngeal swallowing reflex, laryngeal elevation, the presence of penetration or aspiration, and the amount of residue in the vallecular and pyriformis sinuses. Therefore, unlike previous studies, this research may be meaningful in that a machine learning program has been developed that performs similarly compared to physicians in a clinical settings.

This study had several limitations. First, a relatively small sample of VFSSs was used. However, the study showed that the very short time of pharyngeal swallowing reflexes can be measured using machine learning analysis. This is considered to be of sufficient value for a preliminary study. In the future, a more accurate framework using machine learning analysis can be developed through testing with different sample sizes. Second, only the response time of pharyngeal swallowing reflex was analyzed, excluding other spatiotemporal parameters in the oral, pharyngeal, and esophageal phases of the swallowing process. Although interpreting the VFSS is complicated, it is expected that the integration of this framework with similar interpretation methods of the VFSS in clinical situations can be developed in the future.

## Conclusion

This study proposed new automatic measurement of the response time of a pharyngeal swallowing reflex in the VFSS. It can be a clinically useful tool to overcome manually labor-intensive analysis for evaluating the absence or delayed response time of the pharyngeal swallowing reflex in patients with dysphagia. Moreover, it can be useful for clinicians in deciding rehabilitation strategies that may trigger the pharyngeal swallowing reflex, such as the thermal-tactile stimulation.
